# Work from home: bane or blessing? Implications for corporate real estate strategies

**DOI:** 10.1365/s41056-022-00061-3

**Published:** 2022-07-04

**Authors:** Martin Christian Höcker, Yassien Bachtal, Andreas Pfnür

**Affiliations:** Technical University of Darmstadt, Hochschulstraße 1, 64289 Darmstadt, Germany

**Keywords:** Multilocality of work, Workplace preference, Work from home, Corporate real estate, Multilokalität der Arbeit, Arbeitsortpräferenz, Work from Home, Corporate Real Estate

## Abstract

Technological progress and developments in the economy and society are constantly changing the way we work. The ongoing COVID-19 pandemic is accelerating the move towards multilocal working: knowledge workers worldwide have been forced to gain experience of working from home. Based on this experience, they are now in a position to weigh up different places of work and articulate desires for the distribution of working time between home workplace, third places and office.

Previous studies have shown that working from home can have positive effects for corporates in the form of productivity increases. However, it has so far remained open which employees exactly are successful at different workplaces. The aim of the study is to identify clusters with their own workplace distribution based on personal, work-related and real estate characteristics, and to investigate whether the desire for specific workplace distribution promises success.

Identification of the subgroups is done by conducting a hierarchical cluster analysis that includes previously identified personal, work-related and real estate characteristics. The evaluation and interpretation of the cluster solution is based on the desired workplace distribution and identified work success variables. Data from a survey of 2000 German and US knowledge workers is taken into account.

The results of the survey suggest that knowledge workers in Germany and the US have developed a good sense of the workplace in which they can work successfully. At the same time, the decision-makers in the corporates have to decide carefully who should work at which workplace with a view to the corporate’s success. It is also clear that as work becomes more multilocational, real estate resources must play an important role in creating a corporate culture and identity.

## Abstract (de)

Technischer Fortschritt sowie Entwicklungen in Wirtschaft und Gesellschaft verändern die Art des Arbeitens fortlaufend. Die anhaltende Covid-19-Pandemie beschleunigt die Entwicklung hin zu multilokalem Arbeiten: Wissensarbeiter weltweit waren dazu gezwungen, Erfahrung mit dem „Work from Home“ zu sammeln. Diese sind nun in der Lage, aufgrund dieser Erfahrungen über die verschiedenen Arbeitsorte abzuwägen und Wünsche an die Verteilung der Arbeitszeit auf Homeoffice, dritte Orte und Büro zu artikulieren.

Vorangegangene Studien zeigten, dass die Arbeit von zu Hause für Unternehmen positive Auswirkungen in Form von Produktivitätssteigerungen haben kann. Dabei blieb bisher offen, welche Mitarbeiter genau an den verschiedenen Arbeitsorten erfolgreich sind. Ziel der Studie ist es, aufgrund persönlicher, arbeitsbezogener und immobilienbezogener Merkmale Cluster mit eigener Arbeitsortverteilung zu identifizieren und zu untersuchen, ob der Wunsch nach der spezifischen Arbeitsortverteilung Erfolg verspricht.

Die Identifikation der Subgruppen erfolgt anhand der Durchführung einer hierarchischen Clusteranalyse unter Einbezug zuvor identifizierter persönlicher, arbeitsbezogener und immobiliarer Eigenschaften. Die Bewertung und Interpretation der Clusterlösung erfolgt anhand der gewünschten Arbeitsortverteilung und identifizierter Arbeitserfolgsvariablen. Dabei finden Daten aus einer Umfrage unter 2000 deutschen und US-amerikanischen Wissensarbeitern Berücksichtigung.

Die Ergebnisse der Untersuchung legen nahe, dass Wissensarbeiter in Deutschland und den USA ein gutes Gespür dafür entwickelt haben, an welchem Arbeitsort sie erfolgreich arbeiten können. Zugleich müssen die Entscheider in den Unternehmen im Hinblick auf den Unternehmenserfolg mit Bedacht entscheiden, wer an welchem Arbeitsort tätig sein soll. Außerdem wird deutlich, dass die immobiliaren Ressourcen bei zunehmender Multilokalität der Arbeit eine wichtige Rolle beim Schaffen einer Unternehmenskultur und -identität einnehmen müssen.

## Introduction

Even before the onset of the COVID-19 pandemic and the associated spread of working from home, knowledge workers worked from different places than the office in recent years. Whereas work was previously carried out mainly using corporate premises, the so-called “second place”, the use of third places,^1^ such as coworking spaces, has recently also become more widespread in Germany (Bundesverband Coworking Spaces Deutschland 2020). With the onset of the COVID-19 pandemic, work from home was used to an unprecedented extent in order to comply with the required contact restrictions. Work that was traditionally done in the office can now be done in three different places (Gillen 2019). Initial studies indicate that due to the new awareness of employees for the place of work and the advantages of the concepts recognised at corporate-level, all three places of work will continue to retain a significant share in the future spatial distribution of work. It can be assumed that in future, knowledge workers will increasingly weigh-up the location at which they would like to work while taking into account their productivity, job satisfaction and necessity (Pfnür et al. 2021).

On the organisational side, productivity gains have recently been observed as a result of working remotely from home. Pfnür et al. (2021) show an average 14% increase in productivity through work from home was recorded in Germany. However, around 40% of the respondents also stated that they could not perceive any productivity gains or were even less productive working at home than in the office. It can be assumed that employees want to increasingly work from home or from third places (Kniffin et al. 2021 and de Lucas Ancillo et al. 2021) even though these workplaces do not seem suitable from an organisational point of view. For companies, this poses the task of concretely shaping the multilocality of work, also in order to be able to leverage the potentials. But there are a number of unanswered questions. It is still unclear which employees work more successfully at home than in the office. The distribution of working time between the office, home office and third locations cannot yet be quantified either, although this would result in a concrete need for adaptation on the part of the company, for example, through quantitative and qualitative space planning. Furthermore, it must be examined whether the desired distribution of employees’ workplaces also promises work success and is compatible with the company’s goals. This can lead to a conflict of objectives between individual wishes and the overriding corporate goal. After all, previous studies have shown, for example, that not all jobs can be done from home (Dingel and Neiman 2020).

The aim of this paper is to provide a basis for decision-making on the described challenges facing human resources (HRM) and corporate real estate management (CREM). With the help of multivariate analysis methods, subgroups are identified who, based on their personal, work-related and real estate characteristics, will prefer certain workplaces in the future. Finally, the paper assesses whether the desired workplace distribution promises work success for the individual employee and derives management recommendations from the analysis.

The identification of subgroups is carried out by applying a hierarchical cluster analysis. In addition to other variables, factors identified in a previously conducted exploratory factor analysis are taken into account in the analysis. Because previous studies have shown that work success depends on personal, work-related and real estate characteristics of the knowledge workers (Krupper 2015), these determinants are also used in this analysis. The classification and interpretation of the results is based on the given workplace distribution and on further variables on work success at different workplaces identified in a second exploratory factor analysis. The following section examines the theoretical foundation of the change in the working environment, the resulting challenges for corporate management and the influence of individual determinants on workplace preference. This is followed by a description of the data analysis.

## Theoretical background

### Change in the working environment

Changes in the working environment at different workplaces have been driven particularly by technological change. Harris (2015) describes three drivers that are expressions of the development: (1) the organisational adaptation of corporates serves the implementation of collaborative working in order to react continuously to innovation; (2) new requirement profiles for employees, with a demand for technologically affine knowledge workers, which is an expression of the change in the workforce along with the desire within the workforce for flexible working; and (3) technological developments ensure that employees can work smoothly in different locations according to their preferences.

To understand why working from home or third places is attractive to corporates and their employees, it is also necessary to further investigate the benefits of mobile working and teleworking. Even though these terms refer to different forms of work (Bundesministerium für Familie, Senioren, Frauen und Jugend 2017 and 111th United States Congress 2010), the following part of the study will discuss research results on the different forms of work. Although this study is primarily concerned with work from home, telework and mobile work as well as hybrid work and coworking describe multilocal working away from the office and can thus provide important indications and conclusions in this context. Tremblay and Thomsin (2012) have identified greater autonomy, professional and personal development of employees, and a better work-life balance with reduced stress as important benefits of mobile working. In addition, flexibility in daily planning, better organisation of work and reduced commuting times enable a more efficient organisation of the working day. According to Morgan (2004), working away from the office also has advantages from the employee’s point of view by breaking down geographical barriers in the choice of occupation. In addition, he emphasises the cost advantages to be gained from reduced commuting.

In the past, the change in workplace preferences among knowledge workers could be seen in the increase and differentiation of coworking spaces. Thus, special offers for rural areas emerged in cooperation with public institutions or through church organisations (Werther 2021). Nevertheless, a quantitative increase in supply can also be observed (Gauger et al. 2021). The changing proportion of German knowledge workers who do some of their work from home is also evidence of an ongoing balancing process (Statistisches Bundesamt 2021). In the US, a growing trend can be observed: in 2004, 15% of employees regularly worked from home (U.S. Bureau of Labor Statistics 2005); in 2017/18, 25% of employees did so (U.S. Bureau of Labor Statistics 2019). The OECD (2020) reported that in 2015, working from home or at a third place was particularly prevalent in knowledge-intensive occupations and among highly qualified employees.

With the emergence of the COVID-19 pandemic in 2020, a large number of employees changed their place of work and, henceforth, worked from home. The experience of switching to work from home was not exclusively positive. In the short term, both productivity losses from the corporate’s point of view and difficulties on the part of the workforce occurred. For example, the lack of experience with mobile working, the non-existence of the necessary infrastructure, motivational problems and difficulties in organising the working day in a family environment showed that working from home did not prove suitable for everyone (Pfnür et al. 2021; Werther et al. 2021a; OECD 2020; Milasi et al. 2020; Parker et al. 2020).

Thus, many employees, especially those who had previous experience of working from home, found the transition to the home workplace comparatively easier (Milasi et al. 2020). Many employees were able to experience the previously described advantages for the first time and learned to appreciate flexibility, a better work-life balance, or the advantages of saved commuting time (Parker et al. 2020). Corporates and employees experienced a digitalisation boost and prejudices against working from other locations were reduced due to positive experiences. Overall, it was recognised that remote working works and could be an alternative even after the pandemic (Kleinert et al. 2021; Hofmann et al. 2020).

There is widespread agreement that the experience will also serve as a catalyst towards mutlilocal ways of working from a corporate perspective (OECD 2020).

Because the disadvantages of working from home should not be overlooked and because the place of work as described is not suitable for some knowledge workers, an unbalanced switch to solely the home workplace is not expected. Rather, third places, especially coworking spaces, could also be winners of the accelerated development. These could represent a compromise between working from home and office for knowledge workers who lack the prerequisites for successful work at home and yet still desire more flexibility. Thus, a hybrid landscape consisting of all three workplaces could emerge in the future (Mayerhoffer 2021; Werther et al. 2021a). The acceleration also includes the transformation of corporate spaces, which was already initiated before the pandemic, into places of cooperation, exchange and representation (Boland et al. 2020).

### Opportunities and challenges of multilocality of work for corporates

For corporates and the various management disciplines, such as HRM and CREM, work from home presents both opportunities and challenges. For example, in addition to cost benefits and productivity gains, there are also benefits from increased customer service due to wider working hours of the employees, greater geographic proximity of employees to the corporate’s customers, increased agility in responding to emerging challenges and opportunities and the recruitment of new employees from an enlarged talent pool (Morgan 2004). According to Miller (2014), corporates can only achieve the higher workplace occupancy rates they seek for efficiency reasons by using standardised, non-fixed office space and enabling flexible working, including third places and work from home. Thus, workforce multilocality can also provide answers to other challenges.

New demands from employees and corporates are also reflected in the future planning of real estate resources. The change in the workforce, which also goes hand-in-hand with increased employee demands, is countered with measures to remain attractive as a corporate for sought-after employees and to ensure high productivity of highly paid employees. This is also to be achieved by providing the right real estate resource with regard to flexibility and serving the desire for mobile working (Harris 2015). According to the findings of Nanayakkara et al. (2021), workplace consultants and designers believe future offices “would be technology driven, community oriented, sustainability, health and wellbeing focused, smaller in size with satellite offices, such as co-working and office spaces” (Nanayakkara et al. 2021). Also, according to Harris (2015), the office workplace in the future should primarily be a place of collaboration and exchange. In this context, the equipment in these spaces will become increasingly important. Khanna et al. (2013) show that corporates are already using their real estate resources in the adaptation process to convey their corporate culture.

### The determinants of employees’ workplace preference

The influence of personal, work-related or real estate characteristics on the workplace preference of knowledge workers has already been investigated by various studies in the past. Table 1 provides an overview of selected studies.

**Table 1:** Impact of personal, work-related and real estate characteristics on workplace preference

	Authors	Investigated impact of individual determinants on workplace preference
Personal characteristics	Pfnür et al. (2021)	Investigation of the dependence of satisfaction and productivity when working from home on socio-demographic characteristics (age, work experience, income, gender, number of children, relationship status, level of education, occupational stress, loneliness at the home workplace, private and occupational boredom) among German respondents
	Parker et al. (2020)	Investigation of differences in productivity at home between old and young respondents. Mothers perceive greater difficulty in combining work and home life. Women more often express the desire to work from home permanently after the pandemic has subsided
	Horigian et al. (2021)	Demonstration of an increase in loneliness, anxiety and depression as a result of the lockdown in the wake of the COVID-19 pandemic in the US
	Appel-Meulenbroek et al. (2021); Clifton et al. (2019)	Motives for working in third places, such as coworking spaces: isolation when working from home, desire for a sense of belonging and generally the desire for social interaction
	Capdevila (2013); Spinuzzi (2012); Waters-Lynch et al. (2016)	Concentration of coworking space users, for example, in self-employment and freelance work
Work-related characteristics	Pfnür et al. (2021)	Investigation of the dependence of satisfaction and productivity when working from home on the number of work from home days, part-time employment, variety of tasks and demands, and autonomy of planning and decision-making among German respondents
	Dutcher (2012)	Demonstration that routine tasks can be done more productively in the office environment compared to creative tasks
	OECD (2020)	Evidence that highly skilled employees are more likely to be able to cope with working independently and do demanding work in first or third places
	Clifton et al. (2019); Werther et al. (2021b)	Coworking spaces offer an environment for creative problem solving and can thus be a driver for innovation
	Parker et al. (2020); OECD (2020)	Mobile working is not suitable for knowledge workers whose work cannot be done outside the corporate office, at least to some extent. This is especially true for low-skilled workers
	Tremblay and Thomsin (2012); Pabilonia and Vernon (2020)	Influence on the choice of workplace by limiting the autonomy of knowledge workers due to the need for a high proportion of presence in the office for team meetings or with the client, i.e. collaboration
	Spreitzer et al. (2015); Robelski et al. (2019); Tremblay and Thomsin (2012)	Motives for working in third places, such as coworking spaces: deliberate, measured restriction of autonomy, for example, in order to be able to organise one’s self and work better or to have a structured framework for everyday work
	Mokhtarian and Bagley (2000)	In a comparison of the three workplaces, the home workplace promises the highest work-related autonomy while the office workplace is lowest
	Appel-Meulenbroek et al. (2021); Clifton et al. (2019)	Motives for working in third places, such as coworking spaces: possibility of networking in coworking spaces also to win new jobs
Real estate characteristics	Pfnür et al. (2021)	Investigation of the dependence of satisfaction and productivity in the home workplace on real estate determinants (location factors, building- and housing-related factors as well as workplace-related factors) among German respondents
	Parker et al. (2020)	Real estate disadvantages due to a possibly inadequately equipped workplace
	Morgan (2004); Tremblay and Thomsin (2012); Stiles and Smart (2020)	Location advantages through the elimination of commuting times or the possibility of better integration of business trips when working from home. The location advantages described can also be transferred to work in third places
	Robelski et al. (2019); Appel-Meulenbroek et al. (2021)	Motives for working in third places, such as coworking spaces: better working environment with better equipment than at the home workplace. This is expressed both as a possibly more ergonomic equipment of the workplace compared to the home workplace and in the offer of infrastructural space services by the coworking operator
	Clifton et al. (2019); Werther et al. (2021a); Appel-Meulenbroek et al. (2021)	Motives for working in third places, such as coworking spaces: coworking spaces are a representative place for young corporates and self-employed people for their external image. Users also benefit from the comparatively low real estate-related costs and high real estate flexibility associated with coworking spaces
	Frontczak et al. (2012); Kent et al. (2021); Kwon et al. (2019)	Investigations into the influence of the indoor environment quality factors (extent of building space, noise level, visual privacy, cleanliness and maintenance)
	Danielsson and Bodin (2009); Kwon and Remøy (2019)	Investigation of the influence of office layout. Employees perceive higher satisfaction with a cellular structure compared to combi, open and flexible offices

## Methodology and concept of the study

The data on which the statistical analysis is based were collected by the Department of Real Estate and Construction Management at the Technical University (TU) of Darmstadt as part of a research project on work from home. The data were collected by using an online survey among German and American knowledge workers as a longitudinal study with surveys in June, August and October 2020. The characteristics and items taken into account in the analyses were surveyed on metric, simple ordinal or 5‑ or 7‑point ordinal Likert scales. With regard to the Likert scales, a high level of proficiency always indicates a high level of agreement on the part of the respondent while a low score represents a disapproving statement. Regarding the variable household income, it should be noted that respondents were asked to report their income in six salary levels from 0 to > €5000. The first five levels are divided into steps of €1000 each. The last step includes all salaries above that. For example, a respondent who indicated level 3 has a net income between €2001 and €3000.

Out of a total of 2000 respondents, 1159 respondents took part in all three survey waves. Due to the items included and data cleaning, 494 respondents (246 German, 248 US-American) are included in the analysis.

Exploratory factor analyses (EFA) were applied to condense the items collected on Likert scales into factors. Exploratory factor analysis serves to uncover structures within data and to combine individual overlapping variables into factors. This is done by examining the correlation of different items. The factors determined can be used instead of the variables originally investigated. The method is thus particularly well suited to structuring and reducing data. Various extraction methods can be used. The rotation of the solution increases the interpretability of the results (Backhaus et al. 2018). Here, two exploratory factor analyses were carried out. The first analysis serves to derive input factors, which are further used to perform the cluster analysis. The second factor analysis serves to determine factors that describe the success of the respondents at different places of work. They are used to interpret and discuss the cluster results. Both analyses were carried out according to the same principle using the method of principal axis factor analysis and VARIMAX rotation. The number of factors to be considered was determined based on the Kaiser criterion (eigenvalue > 1) (Backhaus et al. 2018; Kaiser 1960) and corresponding content considerations. In order to maintain the original scale level of the items, the factor scores were formed by averaging the items to be combined according to the factor analysis (DiStefano et al. 2009). The standard tests carried out to assess the suitability of the items and the correlation matrix for carrying out an exploratory factor analysis using the MSA criterion according to Kaiser, Meyer and Olkin indicate high suitability of the approach: 0.904 (input factors) and 0.775 (work success factors) at matrix-level and between 0.744 and 0.951 among the items of the input factors, and between 0.662 and 0.866 among the items of the work success factors at item-level (Backhaus et al. 2018; Kaiser and Rice 1974). An overview of the determined factors and associated items is given in Table 11 in the Appendix.

The identification of groups of respondents is done by conducting two cluster analyses: one for German respondents and one for US respondents. The aim of cluster analysis is to identify subgroups from a group of study participants on the basis of their characteristics. The respondents classified in the various groups should be as homogeneous as possible in terms of their characteristics, but the groups should be as heterogeneous as possible in relation to each other. Cluster analysis and the various methods associated with it belong to the explorative procedures. The analysis is divided into three steps: similarity determination, fusion using the selected fusion algorithm and determination of the number of clusters (Backhaus et al. 2018). In this study, the previously identified input factors as well as the variables age, work experience, net household income and commuting time between the corporate office and home are used for clustering. Table 2 provides an overview of the variables taken into account in the cluster analysis and their scales.

**Table 2:** Overview of the variables considered in the cluster analysis

	Variable	Scale	Measurement
Personal	Age	Metric	–
	Work experience	Metric	–
	Household income	Ordinal	–
	Perceived stress with regard to the profession exercised	5‑point-Likert (ordinal)	Determined by EFA
	Perceived loneliness at home workplace	5‑point-Likert (ordinal)	Determined by EFA
	Perceived boredom in private life and job	7‑point-Likert (ordinal)	Determined by EFA
Work-related	Variety of demands and tasks in the job	7‑point-Likert (ordinal)	Determined by EFA
	Planning and decision-making autonomy at work	7‑point-Likert (ordinal)	Determined by EFA
Real estate-related	Technical equipment of the home workplace	7‑point-Likert (ordinal)	Determined by EFA
	Real estate quality and suitability of the home workplace	7‑point-Likert (ordinal)	Determined by EFA
	Commuting time	Metric	–
	Demands on environmental factors in the corporate office	7‑point-Likert (ordinal)	Determined by EFA
	Demands on equipment in the corporate office	7‑point-Likert (ordinal)	Determined by EFA

Similarity between two respondents is determined using squared Euclidean distance. Before the actual analysis is carried out, outliers among the respondents are identified for both countries using the single-linkage method. The exclusion is based on a graphical check of the dendrogram. This procedure has already been successfully proven in the past (Backhaus et al. 2018). Six knowledge workers are excluded from further analysis. As a fusion algorithm, Ward’s method is applied, which has been widely used in practice (Backhaus et al. 2018). Respondents are grouped together in such a way that the dispersion within the groups is increased as little as possible. Thus, the method outputs lead to homogeneous clusters (Backhaus et al. 2018). The determination of the optimal number of clusters can be based on content considerations or on statistical calculations (*stopping rules*). Milligan and Cooper give an overview of the different methods and assess their suitability. Accordingly, Mojena’s test also delivers comparatively good results (Milligan and Cooper 1985) This test compares standardised fusion coefficients ã_i_ based on the coefficients of the fusion overview with a given critical value. If the value is exceeded for the first time, then this is an indicator that the optimal cluster number has been reached with the cluster number of the previous unification stage. Following recommendations from the literature and in order to keep the number of clusters issued within a reasonable range in terms of content, the critical value in this study is set at 2.75 (Backhaus et al. 2018; Milligan and Cooper 1985). Before conducting the cluster analysis, the input variables are standardised by dividing them with the width of the value range due to different value ranges. This is to prevent individual characteristics from having a disproportionately large impact on the distance measurement between two respondents (Miligan and Cooper 1988).

## Results

### Descriptive statistics

The following explanations refer to 243 German and 245 US-American respondents who were taken into account in the final cluster allocation after the outlier adjustment, resulting in a total number of observations of *n* = 488. The desired future workplace distribution of German knowledge workers shown in Table 3 differs from that of the American respondents (Table 4) primarily with regard to the share of third places. Their share is more than twice as high in the US.

**Table 3:** Descriptive statistics of the distribution of desired workplaces of German respondents

Share in %	x̅	s
Work from home	54.6	28.6
Third places	6.2	14.0
Corporate office	39.2	28.0

**Table 4:** Descriptive statistics of the distribution of desired workplaces of US-American respondents

Share in %	x̅	s
Work from home	52.1	29.9
Third places	13.4	21.2
Corporate office	34.5	26.2

Table 5 (German respondents) and Table 6 (US respondents) present the descriptive statistics of the characteristics used for clustering. With regard to the personal characteristics, it is noticeable that the American respondents have on average around 4.0 years more professional experience. In addition, the net household income is higher on average. The perception of boredom among German respondents is subject to smaller fluctuations. The work-related characteristics of occupational autonomy and diversity are on average higher in the US. The same applies to the home workplace-related characteristics of the suitability of the home workplace and its technical equipment. The US respondents’ demands on equipment in the office are also higher than those of their German counterparts.

**Table 5:** Descriptive statistics of the input variables of German respondents

Variable	x̅	Min	Max	s
Age (years)	36.3	18.0	65.0	10.1
Work experience (years)	11.0	0.0	35.0	8.9
Household income	3.5	1.0	6.0	1.3
Perceived stress with regard to the profession exercised	2.7	1.0	4.7	0.9
Perceived loneliness at home workplace	2.5	1.0	5.0	1.0
Perceived boredom in private life and job	2.8	1.0	5.8	1.2
Variety of demands and tasks in the job	5.1	1.0	7.0	1.0
Planning and decision-making autonomy at work	4.9	1.7	7.0	1.2
Technical equipment of the home workplace	5.7	2.3	7.0	1.1
Real estate quality and suitability of the home workplace	5.2	2.0	7.0	1.0
Commuting time (minutes)	25.8	0.0	60.0	14.8
Demands on environmental factors in the corporate office	5.4	3.5	7.0	0.9
Demands on equipment in the corporate office	3.7	1.0	7.0	1.2

**Table 6:** Descriptive statistics of the input variables of US-American respondents

Variable	x̅	Min	Max	s
Age (years)	38.1	20.0	66.0	9.7
Work experience (years)	15.0	1.0	36.0	9.5
Household income	4.7	1.0	6.0	1.4
Perceived stress with regard to the profession exercised	2.6	1.0	5.0	1.0
Perceived loneliness at home workplace	2.5	1.0	5.0	1.1
Perceived boredom in private life and job	2.8	1.0	6.5	1.6
Variety of demands and tasks in the job	5.6	1.0	7.0	1.1
Planning and decision-making autonomy at work	5.4	1.3	7.0	1.1
Technical equipment of the home workplace	6.2	3.0	7.0	0.9
Real estate quality and suitability of the home workplace	5.6	1.6	7.0	1.0
Commuting time (minutes)	24.5	1.0	60.0	13.6
Demands on environmental factors in the corporate office	5.6	3.3	7.0	0.8
Demands on equipment in the corporate office	4.2	1.0	7.0	1.5

With regard to the factors measuring work success at the various workplaces (Table 7 and 8), it is particularly striking that job satisfaction at the home workplace and in the corporate office is on average lower in Germany than in the US. Motivation and focus at the home workplace, on the other hand, are higher in Germany than in the US.

**Table 7:** Descriptive statistics of the work success variables of German respondents

Variable	x̅	Min	Max	s
Job satisfaction working from home	5.3	1.0	7.0	1.3
Productivity working from home compared to the office	4.5	1.0	7.0	1.7
Availability at home	4.5	1.0	7.0	1.2
Motivation and focus working from home	4.4	1.2	7.0	1.3
Job satisfaction in the corporate office	4.7	1.0	7.0	1.2

**Table 8:** Descriptive statistics of the work success variables of US-American respondents

Variable	x̅	Min	Max	s
Job satisfaction working from home	5.8	1.0	7.0	1.1
Productivity working from home compared to the office	4.8	1.0	7.0	1.7
Availability at home	4.7	1.0	7.0	1.2
Motivation and focus working from home	3.9	1.0	7.0	1.5
Job satisfaction in the corporate office	5.4	2.3	7.0	1.0

The description of the clusters obtained from the analysis is carried out in descending order according to the level of the desired work from home share and separately for Germany and the US.

In total, seven clusters per country were delineated based on personal, work-related and real estate characteristics. Table 9 shows the proportions of the different places of work in the clusters.

**Table 9:** Workplace distribution of the different clusters

	Cluster	*n*	Work from Home (%)	Third Places (%)	Corporate Office (%)
German clusters	Senior employees	30	65.5	5.9	28.6
	Skilled workers	22	64.5	5.2	30.3
	Senior managers	14	60.4	3.9	35.7
	Academics	47	54.6	5.2	40.3
	Young professionals	61	53.5	10.0	36.5
	Decision-makers of tomorrow	32	47.5	4.0	48.4
	Under-challenged	37	45.7	5.1	49.2
US clusters	Senior managers	30	70.5	5.0	24.5
	Senior specialists	35	65.0	1.3	33.7
	American dreamers	26	59.6	7.3	33.1
	Nine-to-five clerks	38	54.5	8.3	37.2
	Coworking affine	30	52.6	13.1	34.3
	Office affine	39	46.4	8.8	44.7
	Coworking youngsters	47	29.3	39.1	31.6

Table 10 lists the work success variables of the individual clusters.

**Table 10:** Work success variables of the different clusters

	Cluster	Job satisfaction working from home	Productivity working from home compared to the office	Availability at home	Motivation and focus working from home	Job satisfaction in the corporate office
German clusters	Senior employees	6.2	5.1	4.5	5.2	4.7
	Skilled workers	5.6	4.8	4.1	5.0	4.7
	Senior managers	5.6	4.2	4.5	4.6	4.2
	Academics	5.9	5.0	4.9	4.8	5.2
	Young professionals	5.1	4.6	4.5	3.8	4.3
	Decision-makers of tomorrow	5.0	3.8	4.3	4.1	5.1
	Under-challenged	3.9	3.6	4.3	3.8	4.7
US clusters	Senior managers	6.3	5.1	4.7	4.8	5.6
	Senior specialists	6.2	4.8	4.6	4.4	5.3
	American dreamers	6.4	5.3	4.7	4.2	5.7
	Nine-to-five clerks	5.5	4.1	4.2	3.8	5.2
	Coworking affine	4.6	4.6	4.4	3.4	4.8
	Office affine	6.0	4.2	4.3	4.5	5.6
	Coworking youngsters	5.7	5.6	5.5	2.7	5.8

Table 12 in the Appendix provides an overview of the average expression of personal, work-related and real estate characteristics of the individual clusters. In the following section, the identified clusters of both countries are described in detail. In particular, the characteristics and success variables for which the clusters have a particularly high/low profile are highlighted. Network diagrams are used for a better understanding. In these, the personal (age, professional experience, income, professional stress, perceived loneliness at the home workplace and professional and private boredom), work-related (occupational diversity and autonomy) and real estate-related (technical equipment of the home workplace and suitability of the workplace at home, commuting time and demands on the environmental factors as well as on the technical equipment in the office) characteristics are plotted in a clockwise direction. The bar charts below show the desired work location distribution of the cluster members. In addition, where appropriate for better classification, further variables are discussed that were collected in the context of the surveys but were not taken into account in the cluster analysis.

### German clusters

The cluster of *senior employees* has the highest share of working from home in the future (see Table 9).

**Fig. 1:**
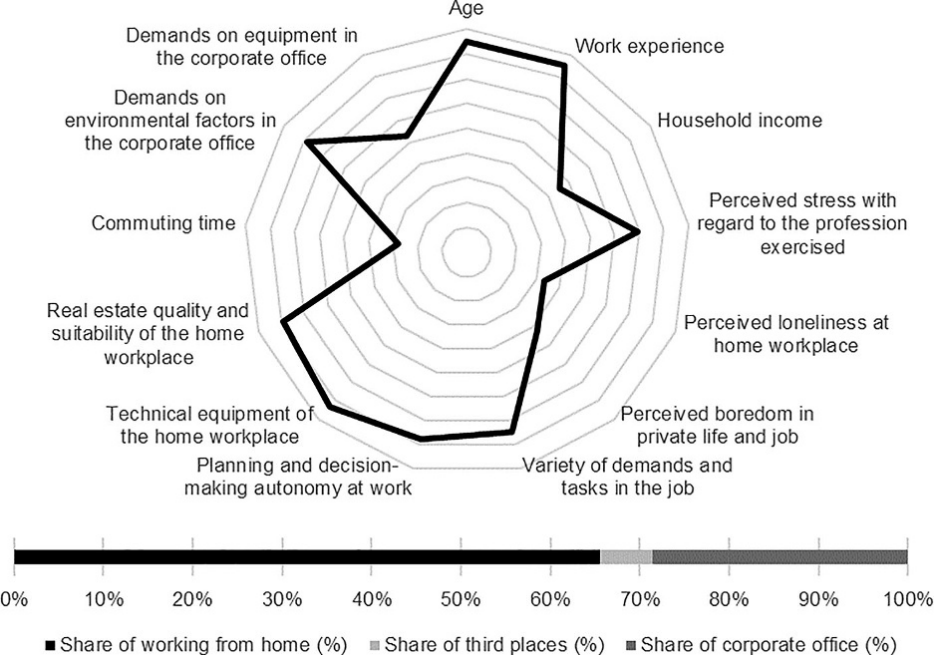
Senior employees (Germany) cluster characteristics

Among the personal characteristics, high age and high professional experience stand out while the household income of the cluster members is particularly low. In addition, only a low level of loneliness and boredom can be observed. The home workplace shows high suitability for working from home regarding quality and equipment. Commuting time of the cluster members is the second lowest among German clusters (see Fig. 1).

Cluster members indicated high job satisfaction, productivity and motivation in the home workplace and, compared to the other German clusters, gave the highest values in all three categories (see Table 10).

Like the *senior employees*, the *skilled workers* want to spend around two-thirds of their working time at home (see Table 9).

**Fig. 2:**
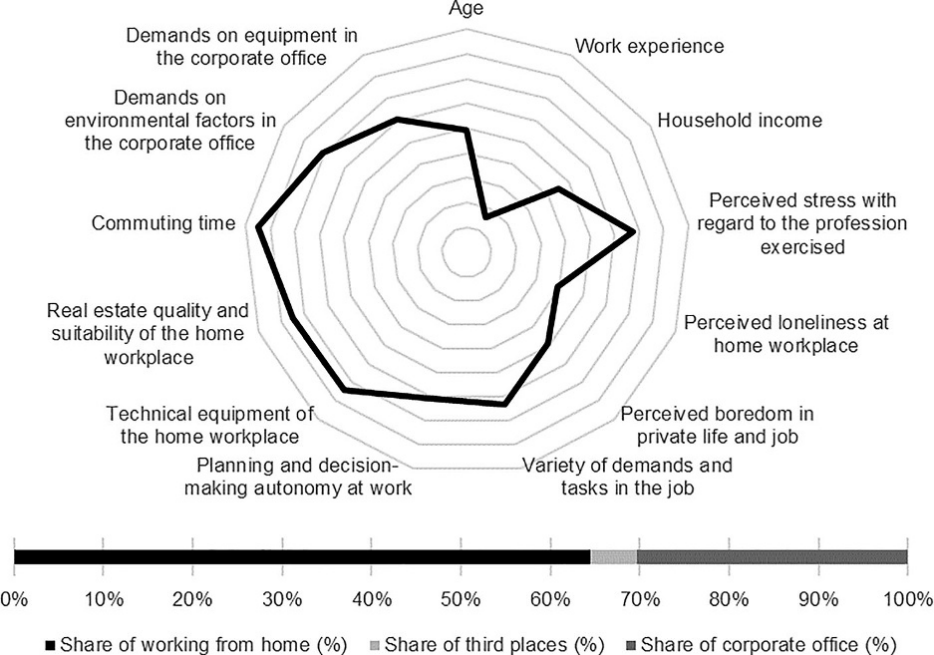
Skilled workers (Germany) cluster characteristics

Among the personal characteristics, the low income and low level of loneliness at the home workplace stand out. In comparison, the cluster members only indicated low levels of work-related autonomy and diversity. The *skilled workers* indicated the highest commuting time and report only low demands on the real estate resource in the office (see Fig. 2).

Among the work success factors, the comparatively high productivity at home, the low additional availability compared to working in the office and the high ability to motivate and focus at the home workplace stand out (see Table 10).

Among the other characteristics surveyed, the comparatively low level of education and the low position in the corporate hierarchy stand out.

The cluster of *senior managers* would like to spend around 60% of their working time at home while the office share is growing to 35% (see Table 9).

Among the personal characteristics of the cluster members, the second highest age and second highest work experience after the *senior employees* stand out. The cluster’s income is the highest of all German groups. The work-related characteristics point to demanding jobs with a wide variety of tasks and a high degree of professional autonomy.

**Fig. 3:**
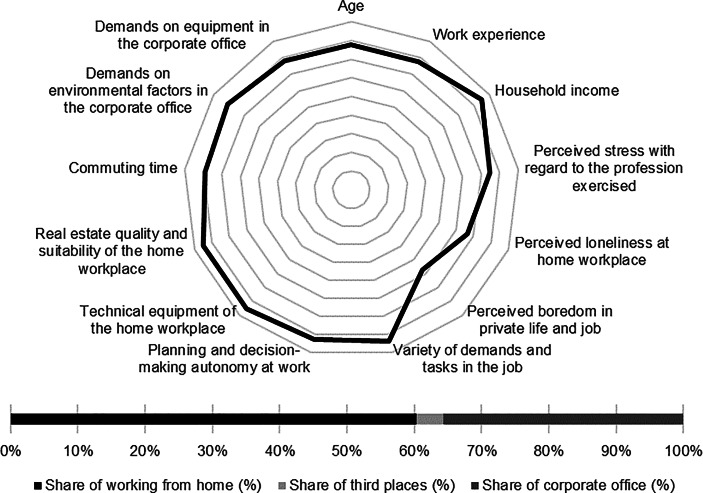
Senior managers (Germany) cluster characteristics

The characteristics related to the home workplace show the highest technical equipment of the workplace and the best suitability of the home for work from home. The commuting time to work is the second highest among German respondents (see Fig. 3).

Among the work success factors, below-average productivity gains stand out for working from home. Satisfaction in the office is the lowest of all German clusters (see Table 10).

*Senior managers* have the highest level of education, the longest working hours and the highest position in the company.

The *academics* cluster wants to work from home around 55% of the time. The office share is over 40% for the first time (see Table 9).

**Fig. 4:**
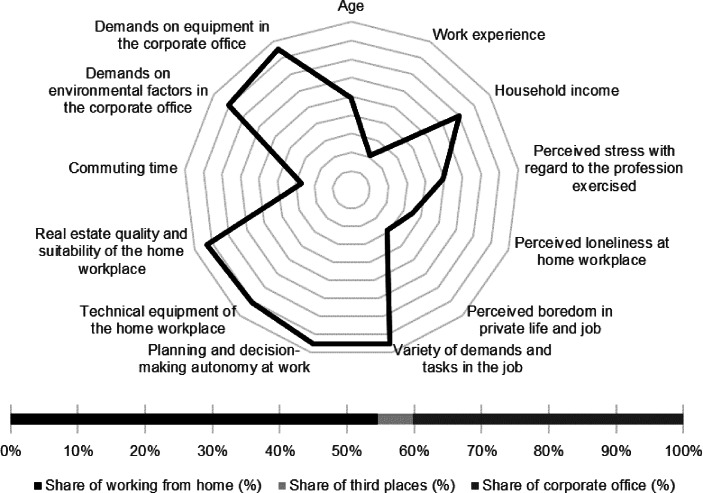
Academics (Germany) cluster characteristics

*Academics* have the second highest income of all German clusters. In addition, they reported the lowest levels of occupational stress, loneliness at home and boredom in their professional and private lives. The jobs they hold are characterised by the highest measured variety of tasks and demands as well as the highest professional autonomy. The cluster members indicated that the home is particularly well-suited for work from home. At the same time, commuting times are particularly low. *Academics* place the highest demands on the equipment of the office workplace (see Fig. 4).

The work success factors show high satisfaction and productivity at home. At the same time, cluster members are exposed to increased availability at the home workplace. Satisfaction with the office workplace is the highest among German respondents (see Table 10).

*Academics* have the second highest level of education after *senior managers*.

*Young professionals* want to spend around 54% of their time at their home workplace. They are the German cluster with the highest share of third locations in the distribution of workplaces. The share accounts for 10% of working time or half a day per week (see Table 9).

**Fig. 5:**
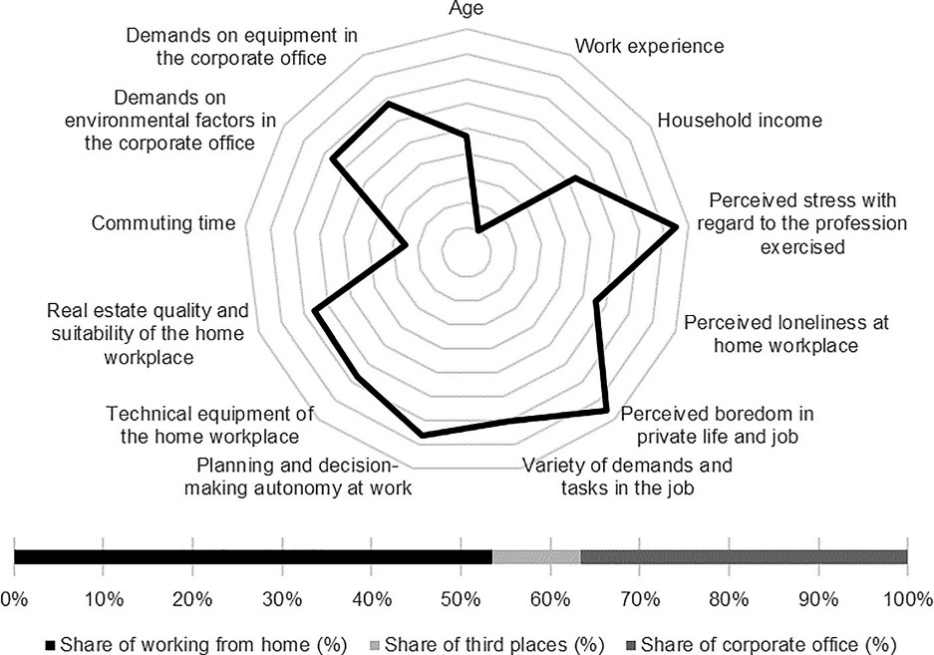
Young professionals (Germany) cluster characteristics

Respondents reported the lowest age and work experience. Among the personal characteristics, the highest level of occupational stress and occupational and private boredom stand out. Among the real estate characteristics, the below-average suitability of the home office and the comparatively low level of technical equipment stand out. At the same time, the respondents indicated the lowest commuting time and articulated only low demands on the environmental factors in the office (see Fig. 5).

Job satisfaction both at home, but even more so in the office, is below average. In addition, respondents indicated the lowest motivation and most distractions at home (see Table 10).

The* young professionals* are the cluster with the lowest number of children at home and the second smallest flats.

The cluster of *decision-makers of tomorrow* would like to work in equal parts in the office and from home. For the first time, the share of office (48.5%) outweighs the share of work from home (47.5%) (see Table 9).

**Fig. 6:**
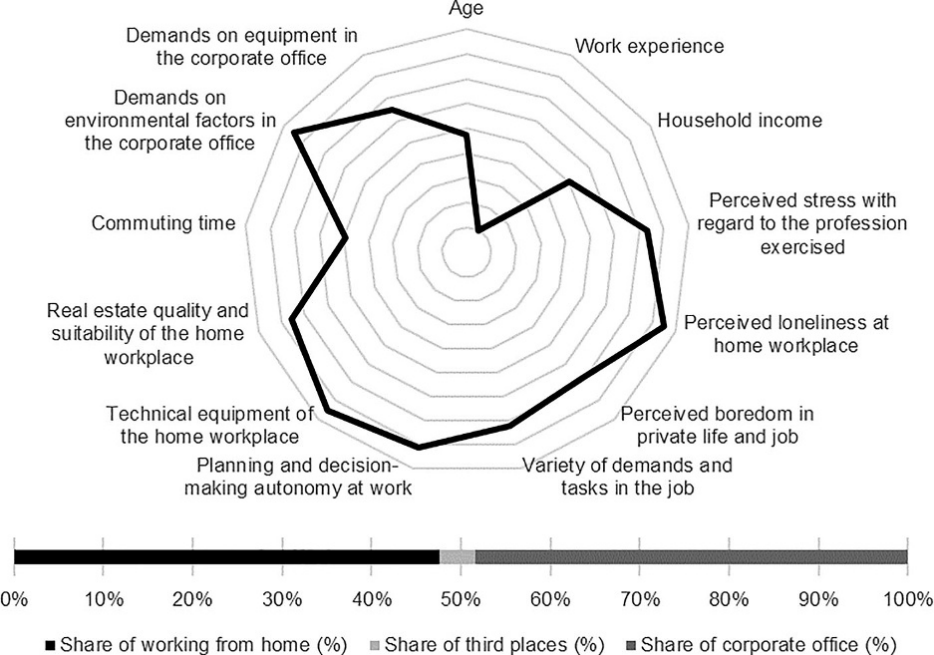
Decision-makers of tomorrow (Germany) cluster characteristics

Like the *young professionals*, the *decision-makers of tomorrow* also indicated a low age and little professional experience. Compared to the *young professionals*, however, they are less affected by job stress and boredom in their professional and private lives. They indicated the highest loneliness of all German respondents at home. Cluster members already have above-average planning and decision-making autonomy. Among the property-related characteristics, the high technical equipment of the home workplace and the highest demand of all German knowledge workers for the environmental factors in the office stand out (see Fig. 6).

The work success factors indicate a below-average suitability of respondents for work from home. Satisfaction as well as productivity and motivation at home are below average among respondents. Satisfaction at the office is the second highest of all German clusters (see Table 10).

The *decision-makers of tomorrow* have the third highest level of education.

The *under-challenged* cluster would like to spend 49% of working time in the office and 46% at home (see Table 9).

**Fig. 7:**
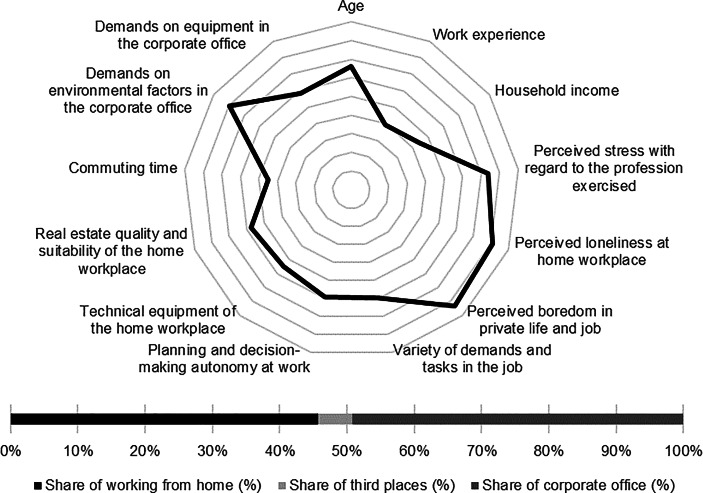
Under-challenged (Germany) cluster characteristics

The *under-challenged* group has the lowest household income of all clusters. In addition, the respondents are affected by comparatively high loneliness at the home workplace and professional and private boredom. The work-related characteristics, i.e. work autonomy and variety, have the lowest levels of all German clusters. The technical equipment of the home workplace and the suitability of the home for work from home also have the lowest values among German respondents (see Fig. 7).

The cluster members indicated the lowest job satisfaction, productivity, motivation and ability to focus at the home workplace (see Table 10).

The *under-challenged* have the least modern office workplace of all German respondents.

### US-American clusters

The cluster of *senior managers* wants to spend 70% of their working time at home (see Table 9).

**Fig. 8:**
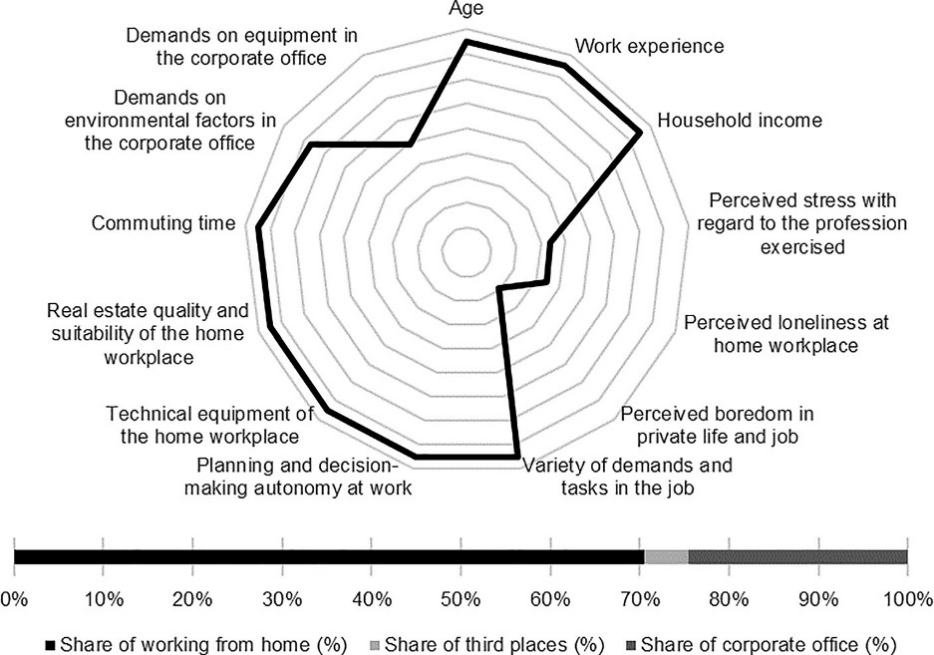
Senior managers (US) cluster characteristics

The cluster includes the oldest and most experienced respondents. *Senior managers* also have the highest income among American respondents. The level of job stress, loneliness at home and boredom in personal and professional life is particularly low while the work-related characteristics of job diversity and autonomy are high. With regard to the technical equipment of the home workplace, the real estate suitability of the apartment for work from home, and the commuting time, the cluster has the highest characteristics of the US clusters. The demands on office equipment are comparatively low (see Fig. 8).

Job satisfaction and productivity at the home workplace are above average. With regard to motivation to work from home, the highest value of all US clusters was measured (see Table 10).

The cluster includes the respondents with the highest level of education and the highest position in their companies.

*Senior specialists* want to spend two-thirds of their working time at home and one-third of their working time in the office (see Table 9).

**Fig. 9:**
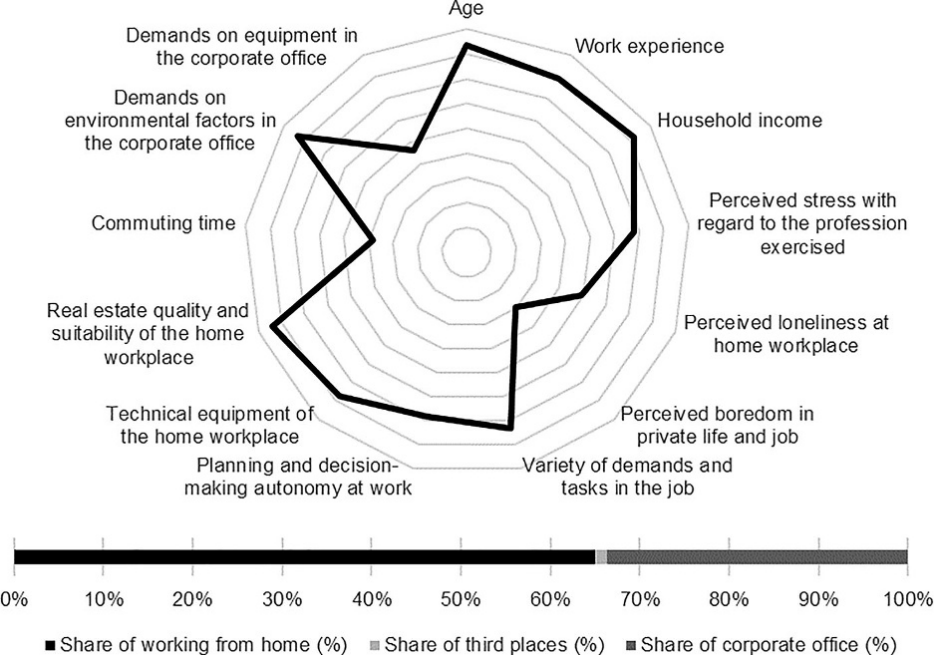
Senior specialists (US) cluster characteristics

The *senior specialist* cluster is the second oldest and second most experienced American cluster. They also have a comparatively high salary. Among the real estate characteristics, the high suitability of housing for work from home is notable. Commuting times are comparatively low and the demands on the environmental factors in the office are comparatively high. The demands on the equipment are very low (see Fig. 9).

The *senior specialists* indicated a high level of job satisfaction at home. Motivation and the ability to concentrate at the home workplace as well as satisfaction in the office are also above average (see Table 10).

The *American dreamers *group wants to spend 60% of their working time at home and one-third of their time in the office (see Table 9).

**Fig. 10:**
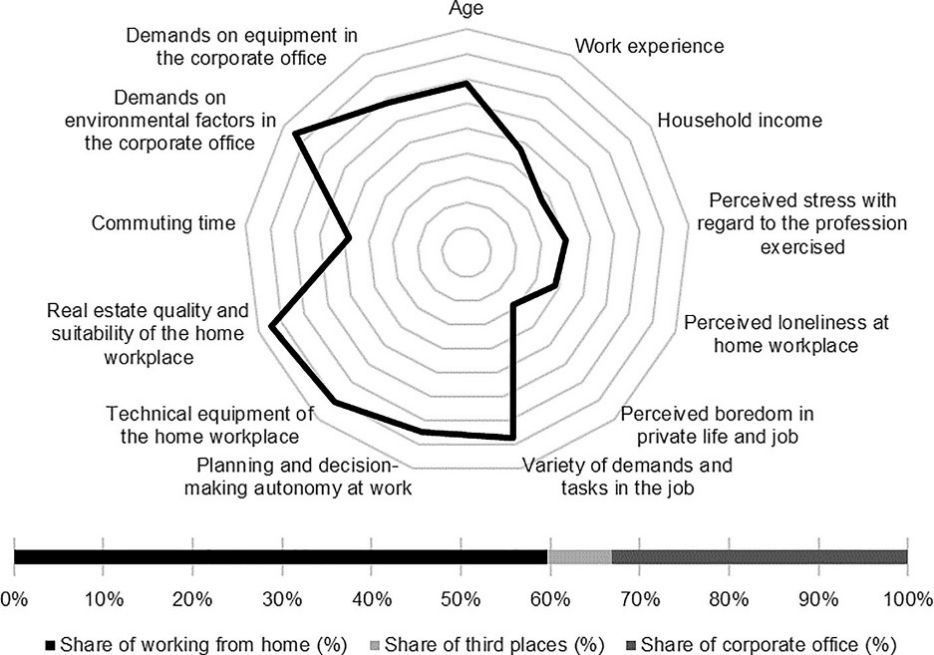
American dreamers (US) cluster characteristics

*American dreamers *have the lowest income of all American clusters. Perceived job stress and loneliness at home are particularly low. With regard to the real estate characteristics, the high suitability of the home workplace and the high demands on the office are noticeable (see Fig. 10).

The *American dreamers *indicated the highest satisfaction of all US clusters at the home workplace. Productivity at the first place is also high. In addition, job satisfaction in the office is the second highest of all clusters (see Table 10).

Among the other variables not taken into account statistically, it stands out that the *American dreamers *have the second lowest level of education and at the same time the longest average working hours.

The *nine-to-five clerks* want to work from home 55% of the time and 37% in the office (see Table 9).

**Fig. 11:**
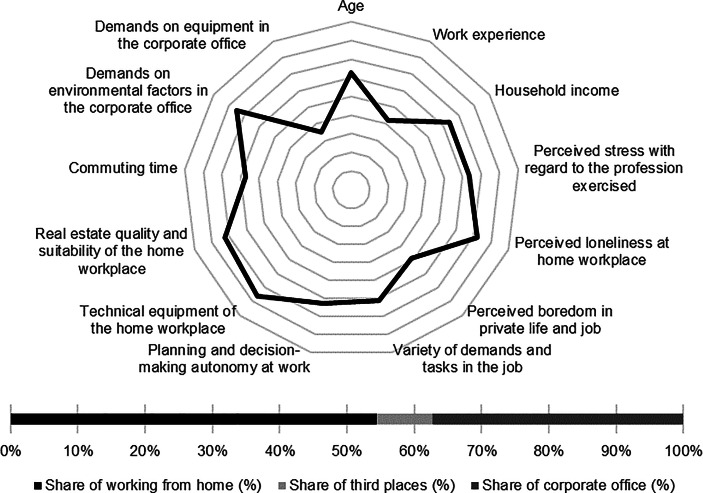
Nine-to-five clerks (US) cluster characteristics

The cluster members have the second highest perception of loneliness at home and a comparatively high level of professional and private boredom. Professional diversity and autonomy are only marginally pronounced. With regard to the suitability of the home for work from home, the respondents gave the second lowest value of all American clusters. The demands on the equipment of the office workplace are the lowest of all American respondents (see Fig. 11).

Cluster members indicated the second lowest level of job satisfaction in the home workplace. Productivity compared to the office workplace is the lowest of all American clusters. However, respondents did not report increased availability at home, such as reduced break times, overtime or working despite being unwell, (see Table 10) and reported an average working time.

The *coworking affine* have the second largest share of coworking time among American employees. They want to spend 13% of their time in third places. More than half of the time they want to work at home and about one-third in the office (see Table 9).

**Fig. 12:**
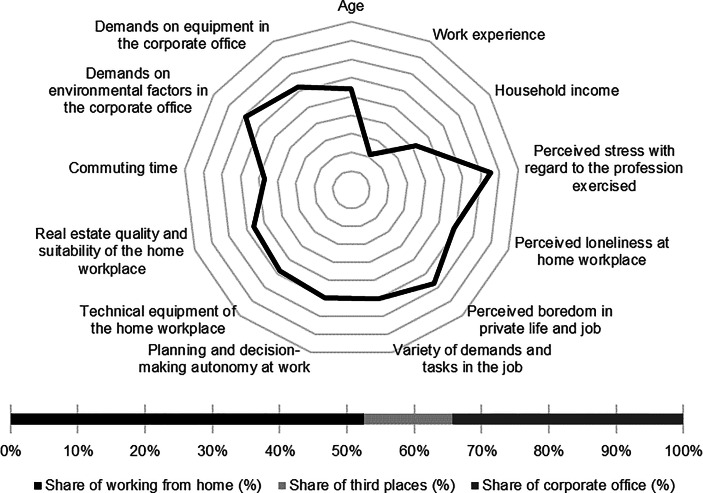
Coworking affine (US) cluster characteristics

The cluster members are comparatively young and inexperienced. The income of the respondents is the second lowest among American clusters and the cluster members experience the second highest professional stress and professional and private boredom. Loneliness at home is also comparatively high. The work-related characteristics of professional autonomy and diversity of tasks and requirements are the least pronounced in the American comparison. The respondents indicated the lowest level of technical equipment and suitability of the home workplace with regard to the property characteristics. The demands on the environmental factors in the office are also the lowest of all American knowledge workers (see Fig. 12).

Among the American clusters, those with an affinity for coworking are those with the lowest satisfaction both at home and in the office (see Table 10).

The distribution of working places of the *office affine* shows that they want to spend the largest proportion of their time in the office compared to other respondents, 45% of their working time at the second place and 46% working from home (see Table. 9).

**Fig. 13:**
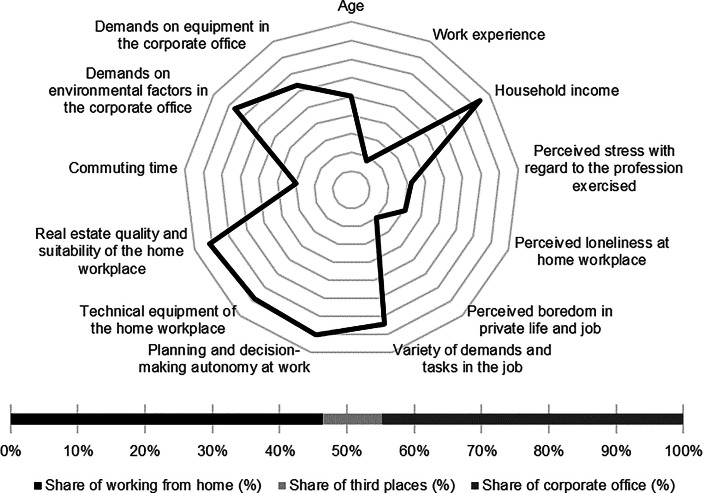
Office affine (US) cluster characteristics

The *office affine* cluster includes the youngest and second least experienced respondents. Their income, on the other hand, is particularly high. The cluster members indicated the lowest affliction of professional stress and loneliness at the home workplace. Professional and personal boredom is the second lowest of all American clusters. Among the work-related characteristics, the high level of autonomy in planning and decision-making should be emphasised. Among the real estate characteristics, only commuting time stands out, the lowest of all US respondents (see Fig. 13).

The cluster of the *office affine* stated the second lowest productivity at home. At the same time, motivation at home is the highest of all American respondents (see Table 10).

The *coworking youngsters* want to spend the largest share of their working time (39%) at third locations. The rest of the time is divided equally between the other two locations (see Table 9).

**Fig. 14:**
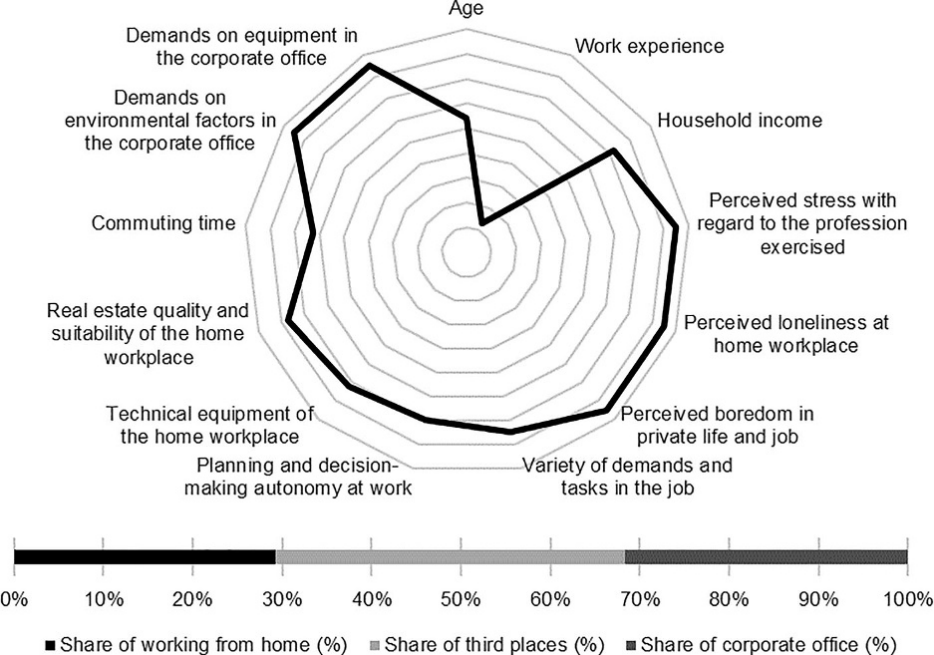
Coworking youngsters (US) cluster characteristics

The respondents are comparatively young and the most inexperienced knowledge workers. Perceived occupational stress, loneliness at home and occupational and private boredom have the highest values across clusters. Among the real estate characteristics, the second highest commuting time and the highest demands on the company office stand out both in terms of environmental factors and equipment (see Fig. 14).

The cluster of *coworking youngsters* indicated the highest productivity at home. At the same time, the motivation to work and the ability to concentrate at the first place is the lowest among the American clusters. Cluster members face increased availability. Job satisfaction in the office is the highest among US knowledge workers (see Table 10).

The *coworking youngsters* have a high level of education and hold comparatively high positions in their company.

## Discussion

### Cluster formation

The clustering of German knowledge workers can be explained based on various developmental strands. With regard to personal characteristics, for example, it can be observed that with a stronger psychographic impact of stress at work, loneliness when working from home and boredom in work and private life, a decreasing share of the home workplace in the desired distribution of workplaces is seen (see clusters *young professionals, decision-makers of tomorrow* and *under-challenged*). With regard to the factors age, work experience and household income as well as the work-related factors, no clear trend can be discerned.

Regarding real estate characteristics, it is clearly recognisable that clusters with high suitability of the home workplace (*senior managers* and *senior employees *as well as *academics*) have the highest share of working from home, whereas, in particular, the two clusters with the lowest suitability of the home workplace (*young professionals* and *under-challenged*) prefer to spend less working time at home. In terms of commuting time, the clusters with by far the longest commuting time (*skilled workers* and *senior managers*) have the second/third largest shares working from home in the future. With regard to the demands on the real estate resource in the corporate office, no clear trend can be discerned.

The work success factors also explain the choice of workplace of the individual clusters. Thus, in the clusters with the highest work from home share, high levels of satisfaction in the home workplace, a good level of motivation as well as productivity advantages in working from home can be observed (*senior employees* and *skilled workers*). In the following clusters, decreasing satisfaction at home, motivation problems and productivity losses at home increase the share of the office and third places. (*senior managers* and *young professionals*) and finally predominate in the remaining clusters (*decision-makers of tomorrow* and *under-challenged*). The cluster of *academics* stands out among the other clusters as the cluster members report above-average success factors in both workplaces. The employees seem to be able to work successfully at all locations and are, therefore, in the middle of all clusters in terms of the proportion of work from home.

Clustering in the US follows similar principles. Among the personal characteristics, there is a tendency that older knowledge workers want to work from home more often. In the US as well, it can be observed that an overall higher psychographic impact of occupational stress, loneliness at home and occupational and private boredom is accompanied by a declining proportion of work from home. *Senior managers* and *American dreamers*, the clusters with the highest and third highest work from home share, respectively, reported only low psychographic strain. At the same time, the burden is particularly high among the cluster of *coworking affine* and *coworking youngsters*. Here, third places seem to offer a good working environment where they can escape loneliness and boredom (compare Appel-Meulenbroek et al. 2021 and Clifton et al. 2019). An exception is the cluster of *office affine*. This cluster has a low level of psychographic stress but has the highest share of working in the office in the future. The cluster members apparently do not flee to the office to escape psychographic stress.

Among the work-related characteristics, it stands out that the clusters with the highest share of working from home have high levels of work-related autonomy and diversity (*senior managers, senior specialists* and *American dreamers*) (compare OECD 2020).

With regard to commuting time, it is noticeable that high commuting times are mostly associated with the desire to work in third places (*coworking youngsters*) or at home (*senior managers*). Low commuting times are associated with higher shares of work in the office (*office affine*). In the US, it can also be observed that high suitability of the home workplace goes hand-in-hand with a stronger desire to work at home (*senior managers, senior specialists* and *American dreamers*). With regard to the demands on the office, no clear trend can be discerned. The cluster with the highest demands is the *coworking youngsters*. The *office affine* cluster is a general exception in the interpretation of property characteristics: it has formulated both an average suitability of the home workplace and an average requirement for the company office. Obviously, the respondents find good working conditions in the office so that the desired proportion of the place of work is high. At the same time, satisfaction with the office is only average. It could probably be increased by upgrading the corporate space.

With regard to the work success factors, the trend can be seen that US knowledge workers tend to work from home if their job satisfaction at the home workplace is high. If satisfaction shifts to the office workplace, then its share and that of third places also increase. The cluster with the lowest satisfaction in the office on the other hand shows an affinity for coworking.

### Comparison between Germany and the US

It was already evident from the comparison of mean values across all respondents in the two countries that third places play a greater role in the US than in Germany. This is also reflected in the cluster results. While there is no cluster in Germany that relies on third places to work, there is one cluster in the US that already prefers third places as their main workplace and another cluster that has an affinity for working at third places. In Germany, third places do not even serve to compensate for long commuting times. The substitution of unsuitable office or home workplace has not taken place yet. In the US, on the other hand, the results of the analysis suggest that coworking, for example, is used to escape of unsuitable home workplace or office or compensate long commuting. US respondents have recognised that high demands on the real estate resource are served in third places. In addition, respondents are consciously looking for a suitable psychographic environment. Apparently, the development towards conscious multilocality of work is already further advanced in the US. German respondents have not yet recognised the advantages of third places of work due to a lack of experience with the place of work. This is in line with the observations of Echterhoff et al. (2018) who observed a low diversity of coworking offers in Germany and called for a further development of the coworking model in order to increase the acceptance of coworking as a place to work.

Furthermore, a higher importance of the office can be seen for German than for US knowledge workers. Five of the German clusters intend to spend around two days or more (> 35%), in the corporate office (79% of all German respondents). Among the US clusters, only respondents from two clusters indicated this (31%). The importance of the corporate office in Germany is underlined by the cluster of *tomorrow’s decision-makers*. These young people, who apparently already have good jobs, want to bear responsibility and also want to do so in the future, rely on the office. Apparently, they see the office as an opportunity to present themselves and to convince the decision-makers of today, the *senior managers*, of their quality. *Tomorrow’s decision-makers* want to be noticed today and they see the corporate office as the stage on which they can present themselves. In the US, on the other hand, there is a cluster that has clearly recognised the advantages of the office. Consequently, it can be deduced that compared to Germany, in the US there is already a better awareness of the various workplaces and the advantages and disadvantages they offer.

### Management implications

#### Listen to your employees

The cluster results clearly show that employees are able to assess for themselves which workplaces are suitable for them. Even if, for example, third places play a subordinate role among German respondents and US-Americans already seem to have a clearer picture of the preferences of the different places of work, they are nevertheless able to differentiate between work and their associated success based on their own characteristics at the home workplace and in the office. Knowledge workers in both countries predominantly prefer workplaces in the future where they can work successfully. The distribution of workplaces also appears to be mostly suitable due to personal, work-related and real estate characteristics. For CREM, this means that the employees of their corporates are the first point of contact for planning the real estate resource. Engaging in dialogue with employees about their workplace preference seems inevitable in the future. The demands articulated by employees can be the basis for the development of real estate strategies.

#### Decide wisely

Even if knowledge workers are well able to decide on an individual level where they want to work successfully, it makes little sense to comply with these wishes without restrictions. Corporates are social entities, and value creation and innovation come from exchanges with one another. The individual goals of the individual respondents cannot be achieved by unreflectively fulfilling all demands either. This becomes clear with the cluster of the *decision-makers of tomorrow*: they prefer to do large parts of their work in the office in order to present themselves to today’s decision-makers and to be noticed. They want to recommend themselves for future tasks. At the same time, *senior managers*, whose job would be to evaluate and train their successors, want to spend most of their working time at home. It becomes clear that the achievement of the individual goals of the first-mentioned group as well as the corporate’s goal of developing and retaining qualified workers in the long term appear questionable if each employee is free to decide to what extent they want to work at different workplaces. Finally, a perceived stagnation of ambitious employees threatens corporates with a brain-drain in the form of migration to other corporates, where the *decision-makers of tomorrow *may assume better development options.

The challenge for HRM and CREM is to optimise the operational space structure while maximising the satisfaction of the needs articulated by the employees and taking into account the business impact contexts such as the need for cooperation and exchange.

#### Use the culture-creating effect of corporate real estate

The survey results clearly show that the future of the working environment is multilocal. As described above, this brings not only advantages but also issues that need to be moderated. If work takes place less in the office in the future and employees are better able to cope in third places and in the home workplace, then this will have an impact on the entire corporate. Not only must smooth and effective work be ensured, but the social component of the corporate must also be preserved in the future. Identification and togetherness can only flourish with difficulty in the home workplace and in third places where employees work in spatial isolation. This makes it all the more important in the future to use corporate real estate in a way that fosters culture. In the time that employees will spend together in their teams at the corporate headquarters, the corporate culture can be communicated. Real estate can provide important impulses for this and transport the desired messages internally and externally.

#### Use multilocality for your purposes

As described above, real estate is becoming increasingly important for communication with corporate stakeholders. At the same time, the expected multilocality of work itself, which is desired by large parts of knowledge workers and which decisively emphasises the importance of real estate, can be used to serve one’s own corporate purposes. In doing so, the focus should not be placed solely on leveraging presumed cost-saving potentials. Rather, the conscious offer of working from home or third places provided by the corporate can also be an instrument for employee acquisition or development. If an employee has the desire to work from home or in a third place and does not find the offer in his or her own corporate, then this could be an argument for changing jobs to a corporate that works in a more hybrid fashion. At the same time, both the German and the US results show that it is, above all, particularly deserving employees who are attracted to the home workplace. Against this background, work from home and work from third places can not only serve to meet real estate needs, but can also be seen as a sign of appreciation as a new status symbol as part of the incentive offer in the war for talents.

#### Shape the multilocal needs and desires of your employees

The German cluster of the *under-challenged* illustrates the opportunities regarding third places of work. The *under-challenged* do not show a high level of satisfaction in either the office or the home workplace. Neither place of work seems to be suitable for the cluster members. The manifestations of professional and private boredom as well as loneliness, and the low suitability of the home workplace indicate that parts of the work could be successfully carried out in third places. However, coworking spaces do not seem to play a role and this is observed in all clusters of German respondents. The *young professionals* show a certain interest in working at third locations and at the same time show low suitability of the home workplace. Here lies an opportunity for the corporates. By offering coworking spaces close to employees’ homes, high commuting times could be compensated and suitable work environments can be used. As the US clusters show, coworking could serve as a substitute for the office or for work from home and, thus, increase employees’ job satisfaction if it is more accepted by the employees. For this to happen, unmet needs must be identified and met. The design of multilocality for employees is, therefore, a valuable key for corporates to manage satisfaction, productivity and costs.

#### The same old tune of qualitative space adjustment in the portfolio

As the previous sections and other studies show, there is considerable need for real estate adaptation on the part of the corporate sector (Pfnür 2020). Work from home plays an important role in all clusters and it is to be expected that it will be used to a considerable extent in the future (Barrero et al. 2021; Pfnür et al. 2021). From a corporate’s point of view, this is an all-important sign as it shows what employees seem to think of the corporate’s space. At the same time, further developments in the US show that corporate offices will continue to have justification for existence that goes beyond mere representation. They continue to play an equal role among the various offerings if, in comparison to these, satisfactory and productive work is possible at the location. Overall, it may be necessary to adjust the quality of the space rather than the quantity. Coworking spaces could make up a larger share of the available space. To maximize the benefits of the real estate resource through the enhancement of exchange and cooperation and as a medium of communication both internally and externally, business space must be of a high quality.

#### Meeting multilocality only works through exchange

Finally, due to the extensive pressure to adapt on the part of the corporates, it should be noted that the change towards a multilocal working world can only succeed in cooperation with all real estate industry players. Such profound changes have an impact on everyone: project developers, investors, the housing industry, urban planning and many more. Increasing multilocality of work changes the demands on the real estate industry as a whole and is a visible sign of the ongoing transformation process. As such, it should continue to be taken into account in future planning.

## Limitations and future research

This paper provides important recommendations for CREM and HRM on how to deal with increasing multilocality of work. However, because the results are based only on surveys of German and US knowledge workers, the findings may not be transferable to other countries. Further research could examine whether the results are transferable and, thus, make an important contribution to the real estate management of multinational corporations. Furthermore, the results could be reviewed after the pandemic—during which the data were collected and which undoubtedly influenced the results—has subsided in order to exclude possible influences of the special situation. In addition, future studies could take into account various other factors to give weight to the factors in terms of their relevance for multilocality. To further sharpen our understanding of the factors influencing multilocality of work, it could also be investigated to what extent the results can be replicated in individual industries or whether industry-specific workplace distributions can be identified. Finally, specific designs of corporate workspaces could also be included in the analysis in order to determine their influence.

## Appendix

**Table 11:** Factors and associated items determined in the EFA

	Factor	Items
Personal	Perceived stress with regard to the profession exercised	1. I feel emotionally drained from my work 2. I feel burnt out by my work 3. I feel drained at the end of the working day
	Perceived loneliness at home workplace	1. I feel lonely in my workplace at home 2. I feel isolated in my workplace at home 3. At my workplace at home, I lack opportunities to socialise at and after work
	Perceived boredom in private life and job	1. I feel bored in my private life 2. I am frustrated in my private life 3. I am not able to concentrate in my private life 4. I am not fascinated by my private life 5. I feel bored in my job 6. I am frustrated in my job 7. I am not able to concentrate 8. I am not fascinated by my tasks
Work-related	Variety of demands and tasks in the job	1. My work requires a wide range of skills 2. My work requires the use of many different skills 3. My work requires the use of sophisticated skills 4. In my work I can use many of my talents 5. In my work I do a lot of different things 6. In my work I am always doing something new 7. In my work I have to work on a variety of tasks 8. My work is very varied
	Planning and decision-making autonomy at work	1. I am free in the timing of my work 2. I can decide for myself the order in which I do my work 3. I can plan my work the way I want to 4. My work allows me to take initiative and act at my own discretion 5. I can make many decisions independently in my work 6. My work gives me a lot of freedom to make decisions
Real estate-related	Technical equipment of the home workplace	1. I have full information and communication technology equipment (computer, printer, etc.) in my home 2. I have a reliable internet connection at my workplace in my home 3. In my home, I have a sufficiently fast internet connection at my workplace
	Real estate quality and suitability of the home workplace	1. All in all, I am very satisfied with the spatial situation of my work at home 2. All in all, I am very satisfied with my housing situation 3. All in all, I am very satisfied with my apartment/property 4. All in all, I am very satisfied with the location of my apartment/property 5. All in all, I am very satisfied with the planning concept of my flat 6. All in all, I am very satisfied with the construction quality of my flat 7. All in all, I am very satisfied with the economy of my housing situation 8. The available rooms (equipment, furniture) support work optimally 9. Creativity is encouraged by the working environment 10. The room acoustics are conducive to work 11. Productivity at work is promoted by the spatial environment 12. I can make undisturbed phone calls/I have sufficient privacy for (spontaneous) phone calls
	Demands on environmental factors in the corporate office	1. I attach great importance to an unobstructed view from the window 2. I attach great importance to fresh, pleasant air 3. I attach great importance to pleasant lighting conditions 4. I attach great importance to a pleasant indoor climate 5. I attach great importance to a low noise level 6. I attach great importance to sufficient space
	Demands on equipment in the corporate office	1. I attach great importance to a height-adjustable desk 2. A couch or armchair is important to me 3. I attach great importance to music at the workplace 4. I attach great importance to good catering facilities (e.g. a high-quality coffee machine) at the workplace
Work success factors	Job satisfaction working from home	1. I am very happy with my home workplace 2. I like working in my home workplace 3. I enjoy working in my home workplace
	Availability at home	1. I take shorter breaks 2. I am available more often 3. I also work, although I would not have felt comfortable enough to work in the office
	Motivation and focus working from home	1. I feel less motivated without my team 2. I am more easily distracted by TV, mobile phone, etc 3. I am more easily distracted by family, child or other people 4. I am more easily distracted by household tasks (e.g. washing, ironing, cooking, etc.) 5. Work and private life get mixed up
	Job satisfaction in the corporate office	1. I am very satisfied with my office workplace 2. I like working at my office workplace 3. I enjoy working in my (company) office

**Table 12:** Personal, work-related and real estate characteristics of the different clusters

	Cluster	*n*	Age	Work experience	Household income	Perceived stress with regard to the profession exercised	Perceived loneliness at home workplace	Perceived boredom in private life and job	Variety of demands and tasks in the job	Planning and decision-making autonomy at work	Technical equipment of the home workplace	Real estate quality and suitability of the home workplace	Commuting time	Demands on environmental factors in the corporate office	Demands on equipment in the corporate office
German clusters	Senior employees	30	50.3	24.4	3.1	2.6	1.7	2.1	5.2	5.1	6.3	5.5	19.9	5.6	3.0
	Skilled workers	22	32.3	7.5	3.0	2.6	1.9	2.4	4.5	4.1	5.7	5.3	46.4	5.1	3.4
	Senior managers	14	46.8	22.6	5.1	2.8	2.9	2.7	5.7	5.4	6.4	5.9	43.6	5.7	4.1
	Academics	47	32.3	8.8	4.3	2.0	1.8	1.6	5.8	5.5	6.1	5.7	19.6	5.7	4.5
	Young professionals	61	31.2	6.1	3.4	3.1	2.5	3.7	4.9	5.0	5.3	4.8	18.6	4.8	3.7
	Decision-makers of tomorrow	32	31.4	6.1	3.3	2.8	3.6	3.1	5.0	5.3	6.4	5.3	29.9	6.0	3.6
	Under-challenged	37	40.9	13.2	3.0	2.8	3.4	3.6	4.3	4.1	4.5	4.3	27.8	5.6	3.3
US clusters	Senior managers	30	47.9	26.7	5.6	1.7	1.7	1.6	6.5	6.3	6.8	6.0	36.4	5.5	3.6
	Senior specialists	35	47.3	25.2	5.4	2.9	2.3	2.1	5.7	5.2	6.3	6.0	19.4	5.8	3.4
	American dreamers	26	39.9	16.5	2.9	1.9	1.9	2.0	6.0	5.7	6.5	6.0	22.9	5.9	4.7
	Nine-to-five clerks	38	37.4	15.2	4.4	2.8	3.1	3.0	4.9	4.9	6.2	5.3	26.2	5.4	2.8
	Coworking affine	30	33.2	9.8	3.2	3.2	2.6	3.9	4.9	4.7	5.0	4.3	22.6	5.0	4.3
	Office affine	39	31.3	8.6	5.5	1.7	1.6	1.6	5.8	6.0	6.3	5.8	16.3	5.5	4.4
	Coworking youngsters	47	33.1	7.4	4.9	3.5	3.5	4.7	5.8	5.4	5.9	5.6	28.3	6.0	5.6

## Footnotes

1 The definition of third places as public places of social coexistence and intensive communication away from one’s own home (first place) and workplace (second place) goes back to Oldenburg and Brissett (1982). Oldenburg and Brissett also observed that third places are used for work. In this study, this includes not only cafés, restaurants, lounges in train stations or airports, but also coworking spaces. Morisson (2019), referring to Oldenburg and Brissett, defines coworking spaces as a “combination of the second [of labour] and third place”.
